# Tumor progression locus 2 (TPL2) in tumor-promoting Inflammation, Tumorigenesis and Tumor Immunity

**DOI:** 10.7150/thno.45848

**Published:** 2020-07-09

**Authors:** Lucy Wanjiru Njunge, Andreanne Poppy Estania, Yao Guo, Wanqian Liu, Li Yang

**Affiliations:** 1 The Key Laboratory of Biorheological Science and Technology, Ministry of Education, College of Bioengineering, Chongqing University, Chongqing 400044, China.; 2 The 111 Project Laboratory of Biomechanics and Tissue Repair, College of Bioengineering, Chongqing University, Chongqing 400044, China.

**Keywords:** Tumor progression locus 2, tumor-associated inflammation, tumorigenesis, tumor immunity, TPL2-adaptor function

## Abstract

Over the years, tumor progression locus 2 (TPL2) has been identified as an essential modulator of immune responses that conveys inflammatory signals to downstream effectors, subsequently modulating the generation and function of inflammatory cells. TPL2 is also differentially expressed and activated in several cancers, where it is associated with increased inflammation, malignant transformation, angiogenesis, metastasis, poor prognosis and therapy resistance. However, the relationship between TPL2-driven inflammation, tumorigenesis and tumor immunity has not been addressed. Here, we reconcile the function of TPL2-driven inflammation to oncogenic functions such as inflammation, proliferation, apoptosis resistance, angiogenesis, metastasis, immunosuppression and immune evasion. We also address the controversies reported on TPL2 function in tumor-promoting inflammation and tumorigenesis, and highlight the potential role of the TPL2 adaptor function in regulating the mechanisms leading to pro-tumorigenic inflammation and tumor progression. We discuss the therapeutic implications and limitations of targeting TPL2 for cancer treatment. The ideas presented here provide some new insight into cancer pathophysiology that might contribute to the development of more integrative and specific anti-inflammatory and anti-cancer therapeutics.

## Introduction

Tumor progression locus 2 (TPL2, also known as cancer Osaka thyroid (Cot) and MAP3K8) is a serine/threonine kinase that was initially identified as an oncogene and a target for provirus integration 1. The TPL2 gene encodes two proteins, Tpl-p58 and Tpl-p52 that arise from alternative translational initiation at methionine1 (M1) and methionine30 (M30), respectively 2, 3. Structurally, the TPL2 protein contains the N-terminal domain (amino acids 29) that might negatively regulate TPL2 stability and positively regulate its cell transformation capabilities; the serine/threonine kinase domain at the center of the protein that primarily targets MAPK kinase 1/2 (MEK1/2); the carboxy (C)-terminus that functions to inhibit kinase activation and negatively regulate TPL2 stability and transformation capability 1, 4. The C-terminus is proposed to modulate TPL2 kinase activity by folding back onto the kinase domain and by promoting TPL2 protein proteolysis at the degron sequence (amino acids 435-457) 1, 4. Consequently, truncation of the C-terminus is reported to increase TPL2 kinase activity and stability, as well as activating its transformation potential 1, 4. In addition, TPL2 can function as a kinase or as an adaptor protein. In physiological conditions, TPL2 forms a stoichiometric complex with a small fraction of cellular NF-κB1 p105 and the majority of cellular A20 binding inhibitor of NF-κB 2 (ABIN-2) 4. NF-κB1 p105, a precursor for the NF-κB p50 subunit, functions as a stabilizer and competitive inhibitor of TPL2 kinase, while the ABIN2 protein co-dependently stabilizes TPL2 protein 1, 4, 5. TPL2 kinase conveys various intra-cellular and extra-cellular stimuli to effector proteins that regulate the expression of pro-inflammatory cytokines, chemokines, enzymes and growth factors involved in inflammatory and immune cell recruitment, differentiation and activation 1, 5, 6.

The idea that TPL2-driven inflammation is linked to cancer is not entirely a new concept. In 2014, the Hye Won Lee group demonstrated that TPL2 induces castration-resistant prostate cancer progression and metastasis through the activation of the inflammatory CXCL12/CXCR4 and FAK/Akt signaling 7. Accordingly, Li Xinli et al. proposed that TPL2 kinase drives hepatic pro-tumorigenic inflammation and promotes hepatocellular carcinoma (HCC) development through the upregulation of pro-inflammatory cytokines interleukin (IL)-1β, IL-18, monocyte chemoattractant protein 1 (MCP-1), inflammasome (NACHT, LRR and PYD domain-containing protein 3, NALP3) and endoplasmic reticulum (ER) stress 8. TPL2 expression and activity are also implicated in the progression of several inflammatory-associated cancers, including pancreatic cancer, liver cancer and colitis-associated cancer 4, 6, 9. In these cancers, TPL2 activity contributes to inflammatory and immune cell recruitment and the production of cytokines, chemokines, growth factors and enzymes that propagate tumor initiation, tumor promotion, tumor progression and immune responses 4, 6, 9.

Although there is extensive documentation of TPL2 role in inflammation, cancer and immune diseases, the importance of TPL2-driven inflammation in tumorigenesis and tumor immunity, as well as the underlying mechanisms that drive these processes, are not fully appreciated and understood 1, 5, 6, 9, 10. Here, we attempt to dissect the molecular mechanisms in which TPL2 connects inflammation to tumorigenesis as well as tumor immunity by focusing on recent insight on the TPL2 function. We also discuss some controversial findings that propose the anti-inflammatory and anti-tumorigenic functions of TPL2, and their relevance to TPL2-driven inflammation and tumorigenesis. Finally, we discuss the prospects of TPL2 signaling as a therapeutic and complementary target for cancer treatment.

## TPL2 and tumor-promoting inflammation

### TPL2-directed inflammatory signaling

**Figure 2:**
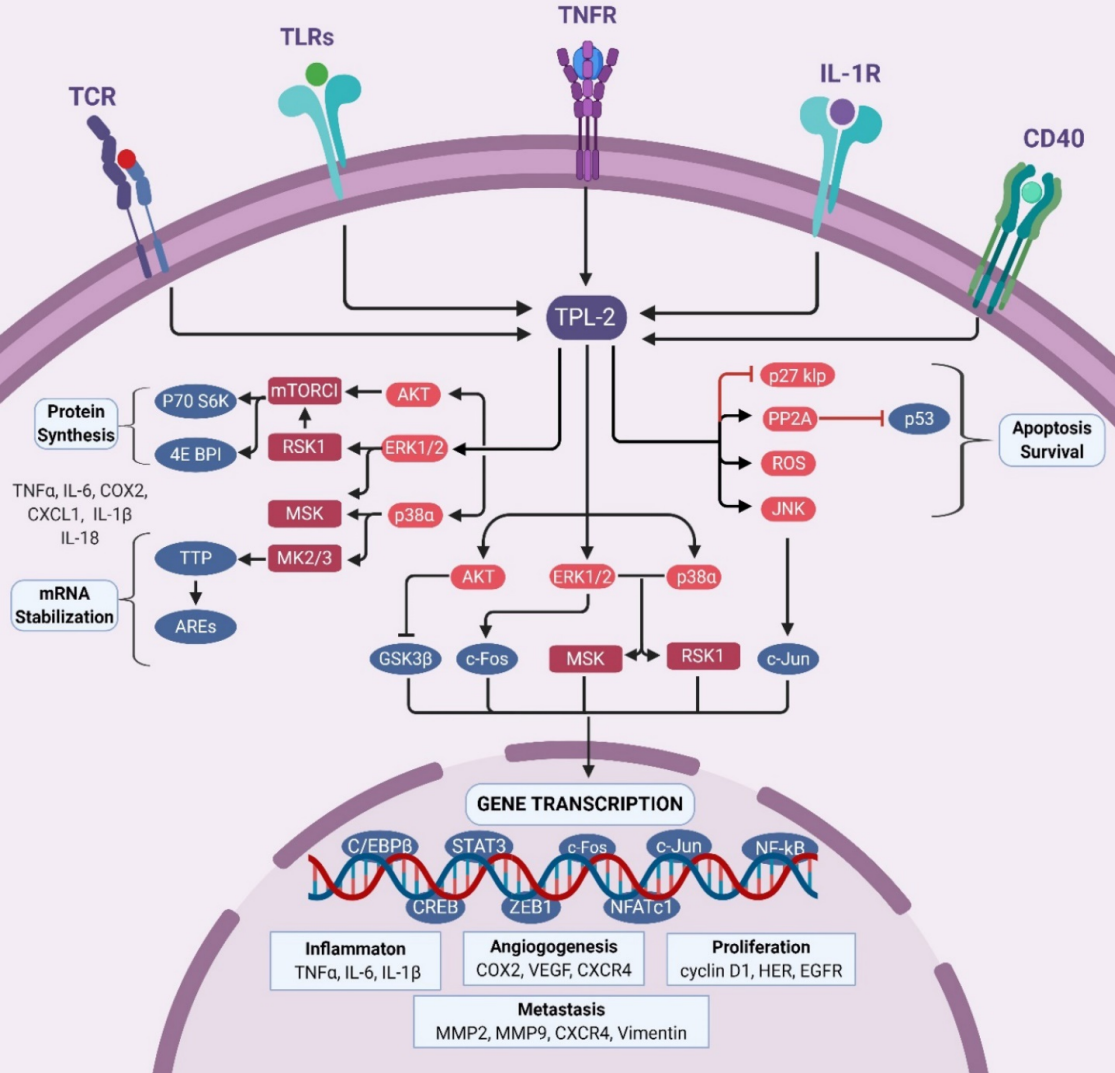
** The TPL2 signaling**. TPL2 signaling pathway is activated following the stimulation of several receptors including TNFR, IL-1R, TLRs, CD40 and TCR. Activated TPL2 subsequently activates MAPKs signal: ERK1/2, p38α, JNK, and Akt that regulate the activation of several transcription factors: NF-κB, AP1, STAT3, C/EBPβ, CREB, ZEB1 and NFATc1. These transcription factors induce the expression of various genes such as TNFα, IL-6, IL-1β, COX2, VEGF, CXCR4, cyclin D1, HER, EGFR, MMP2, MMP9 and vimentin that are involved in inflammation, cell proliferation and survival, angiogenesis and metastasis. In addition, TPL2 promotes cell survival by inhibiting p27kip expression, deactivating p53 through PP2A activity and through the regulation TACE expression. TPL2 signaling is also involved in mRNA stabilization and protein translation. In the cytoplasm, TPL2-activated ERK1/2 and Akt regulate the mTORC1/S6 signaling pathway that modulates the protein translation of inflammatory factors including TNFα, COX2, CXCL1, IL-1β and IL-18. Activated p38 might also promote mRNA stabilization by activating the MK2. Moreover, activation of RSK1 and MSK1 by ERK1/2 and p38α can promote transcription factor activation, mRNA stabilization and translation. (TPL2: tumor progressive locus 2; TNFR: tumor necrosis factor receptor; IL-1R: interleukin 1 receptor; TLRs: toll-like receptors; CD40: cluster of differentiation 40; TCR: T cell receptor; ERK1/2: extracellular signal-regulated kinases 1/ 2; JNK: c-Jun N-terminal kinase; Akt: protein kinase B; MAPKs: mitogen-activated protein kinases; mTORC1: mammalian target of rapamycin complex 1; NF-κB: nuclear factor kappa-light-chain-enhancer of activated B; AP1: activator protein 1; STAT: signal transducer and activator of transcription; C/EBPβ: CCAAT-enhancer-binding proteins-β; CREB: cAMP response element-binding protein; ZEB1: zinc finger E-box-binding homeobox 1; NFATc1: nuclear factor of activated T-cells, cytoplasmic 1; TNF: tumor necrosis factor receptor; IL: interleukin; COX2: cyclooxygenase 2; CXCL: CXC-chemokine ligand; CXCR: CXC-chemokine receptor; VEGF: vascular endothelial growth factor; EGFR, epidermal growth factor receptor; MMP: matrix metalloproteinase; Pin1: peptidyl-prolyl cis/trans isomerase; HER: human epidermal growth factor receptor; EGFR: epidermal growth factor receptor; RSK1: ribosomal protein S6 kinase 1; MSK1: mitogen- and stress-activated kinase 1; MK2: MAPK activated protein kinase 2).

TPL2 is quickly and transiently activated by viral and bacterial infection, oxidative stress, necrotic products, harmful chemicals, pro-inflammatory cytokines and pathogen-associated or damage-associated molecular patterns (PAMPs and DAMPs, respectively) 1, 9. These agents activate various inflammatory receptors including tumor necrosis factor receptor (TNFR), the cluster of differentiation 40 (CD40), interleukin 1 receptor (IL1R), pattern-recognition receptors (PRRs) such as Toll-like receptors (TLRs) and T cell receptor (TCR) 9. These activated receptors convey the signal to signal transducers and amplifiers, such as interleukin 1 receptor-associated kinase (IRAK), lymphoma-associated myeloid differentiation 88 (MyD88) and TNF receptor-associated factor (TRAF), which lead to the activation of the IκB kinase (IKK) complex, IKKα/IKKβ/IKKγ 1, 6, 9. The IKKβ subsequently phosphorylates NF-κB1 p105 at serine 927 and serine 932, tagging it for proteasome degradation, which leads to the subsequent release, phosphorylation and activation of the TPL2 kinase 1, 5, 6. TPL2 then activates a myriad of effector molecules, including extracellular signal-regulated kinases 1 and 2 (ERK1/2), p38α, c-Jun N-terminal kinase (JNK) and protein kinase B (PKB, also referred to as Akt), which direct pathways and transcription factors responsible for the induction of cytokines, chemokines, proteinases, growth factors, and oxidative stress (Figure 2) 11-13. Some of the TPL2 activation seen in cancers may be due to the mutation or dysregulation of the signaling components that activate TPL2 kinase 14. For example, activation of MyD88 gain of function mutation such as Leu265Pro (L265P) within the MyD88 Toll/interleukin 1 receptor (TIR) domain, results in the activation of TPL2 upstream ERK1/2 activation in lymphoid neoplasms 15.

TPL2-regulated genes include cytokines (TNF, IL-1α/β, IL-6, IL-17A, IFNγ and IL-10), chemokines (such as IL-8, CXC-chemokine ligand 1 (CXCL1), CC-chemokine ligand 3 (CCL3/MIP1α), CCL2/MCP1 and CCL5/RANTES), growth factors (granulocyte-macrophage colony-stimulating factor (GM-CSF) and vascular endothelial growth factor (VEGF)), proliferative proteins (peptidyl-prolyl cis/trans isomerase (Pin1), cyclooxygenase 2 (COX2) and cyclin D1) and matrix metalloproteinase (MMP2, MMP3, MMP9 and MMP13) 1, 6, 10. TPL2 kinase is also implicated in the post-transcriptional regulation of TNFα, IL-6, COX2, IL-1β and IL-18 through multiple mechanisms, including nucleoplasmic transportation, mRNA stabilization, translation and protein secretion 5, 16-18. The TPL2 post-transcriptional modulation of inflammatory factors offers a strategic advantage that controls the net effect of inflammatory output, thereby providing rapid inflammatory responses that might potentiate the pro-tumorigenic inflammatory environment 17, 18. However, the implication of TPL2-mediated translational control in tumor-associated inflammation and tumorigenesis has not yet been determined and warrants further studies.

The TPL2-regulated cytokines and chemokines further promote the recruitment of inflammatory cells such as macrophages, neutrophils and lymphocytes, which contribute to the inflammatory microenvironment 1, 10. These cells produce a plethora of inflammatory factors such as TNFα, IL-6, CXCL12 and CXCL1 that facilitate cell proliferation, cell survival, angiogenesis and metastasis through the activation of inflammatory and oncogenic pathways 6, 10. Some of the oncogenic pathways regulated by TPL2 include mitogen-activated protein kinase (MAPK), nuclear factor-kappa light-chain enhancer of activated B cells (NF-κB), activator protein 1 (AP-1), signal transducer and activator of transcription 3 (STAT3), mechanistic target of rapamycin (mTOR) and CCAAT/enhancer-binding protein β (C/EBPβ) (Table 1) 6, 10. Hence, TPL2-mediated inflammatory signaling creates a positive feedback loop that demonstrates a plausible mechanism in which cancer cells and other cells manipulate the tumor microenvironment to promote pro-tumorigenic inflammation 19. By regulating various inflammatory factors, transcription factors and post-transcription mechanisms, TPL2 controls a hub of pro-tumorigenic inflammatory signaling that might play a decisive role in determining tumor-associated inflammation and tumor fate.

### The function of TPL2 in tumor-promoting inflammation

Chronic inflammation from persistent viral and bacterial infection, autoimmunity and carcinogens are suggested to augment core cellular and molecular adaptations that precede oncogenic activities such as mutation, genomic instability, tumor promotion and angiogenesis 20, 21. Accordingly, well known human carcinogens, such as carcinogen N -methyl- N′-nitro- N -nitrosoguanidine (MNNG) and arsenite, are reported to activate TPL2 kinase activity 19, 22. Activated TPL2 then mediates the activation and progression of several oncogenic processes, including cell proliferation, EMT progression, angiogenesis and metastasis through the activation of NF-κB, AP-1, STAT3, and C/EBPβ signaling pathways 19, 22.

Moreover, the tumorigenic pathogen Helicobacter pylori, associated with gastric cancer and mucosa-associated lymphoid tissue (MALT), is reported to increase host susceptibility to infection, inflammation and tumorigenesis through TPL2 dependent pathway 20, 23. TPL2 signaling is also directly implicated in viral-induced malignant transformation and tumor progression. For example, the oncogenic Epstein-Barr virus (EBV), linked to Hodgkin's lymphoma and EBV-associated nasopharyngeal carcinoma, is shown to activate the TPL2 kinase, which then modulates viral lytic replication, NF-κB and AP-1 activation, inflammation and tumor progression 24, 25. The identified role of TPL2 signaling as a target and modulator of pathogen-driven tumorigenesis needs further investigation.

Several studies have implicated TPL2 in controlling inflammatory responses in non-hematopoietic cells that drive the pathogenesis of chronic inflammatory diseases, including neuroinflammation, lung inflammation, obesity-associated chronic inflammation, pancreatitis, hepatic inflammation and vascular inflammation 26-30. Moreover, TPL2 is also implicated in the onset and progression of several inflammatory-related autoimmune diseases, including diabetes, multiple sclerosis (MS), rheumatoid arthritis (RA), inflammatory bowel disease (IBD), and thrombocytopenia (ITP) 31-35. Collectively, these studies propose the importance of TPL2 kinase in propagating chronic inflammation that might drive malignant transformation through the production of inflammatory cytokines and the activation of various inflammatory and oncogenic signaling pathways.

## TPL2 and tumorigenesis

**Figure 3:**
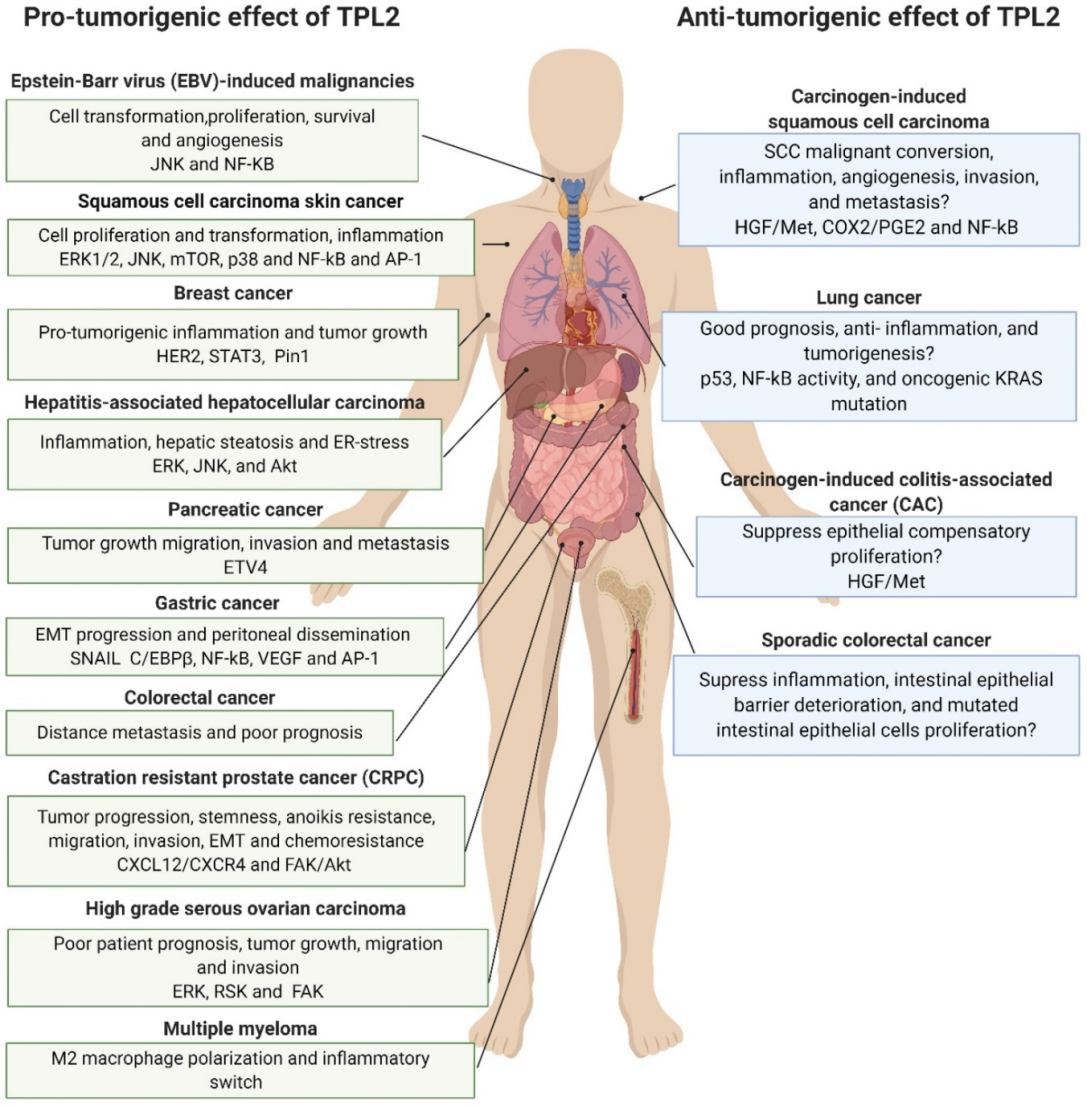
** Pro- and anti-tumorigenic effects of TPL2 signaling**. The TPL2 kinase activity functions as a potent tumor promoter in most cancers, where it is predominantly associated with increased inflammation, malignant transformation, tumor growth, stem cell acquisition, angiogenesis, metastasis and poor prognosis. However, suppressed TPL2 expression is also reported in some cancers like lung cancer and its ablation is associated with increased tumorigenesis in experimental skin cancer and intestinal cancer. It is worth noting that increased tumorigenesis in TPL2 suppressed carcinogen-induced squamous cell carcinoma, carcinogen-induced colitis-associated cancer, lung cancer and sporadic colorectal cancer is mostly dependent on the presence of tumor promoting conditions such as inflammation, tissue damage and NF-κB signaling. (The figure was created with BioRender.com, ©BioRender 2020).

Initially, TPL2 was identified as a target for provirus integration in Moloney murine leukemia virus (MoMuLV)-induced T cell lymphomas and mouse mammary tumor virus (MMTV)-induced mammary carcinomas in mice 4, 5. The viral insertion induced the generation of a truncated form of the TPL2 protein, which portrays increased activity and stability with enhanced transformation potential, illustrating the importance of increased TPL2 kinase activity in tumorigenesis 4. Although TPL2 mutations are rare in human cancers, there is some evidence of mutations that induce TPL2 signal amplification and constitutive kinase activity occurring in breast cancer and lung adenocarcinoma, raising the possibility that TPL2 C-terminal might be a target of mutation in some cancers 5, 36, 37. However, the number of tumors with TPL2 overexpression and activation is much more significant than the subfraction of tumors with confirmed mutations, suggesting that TPL2 overexpression and activation are the main events associated with increased tumorigenesis 5. TPL2 overexpression and increased activity are associated with poor prognosis and the progression of several human cancers including skin cancer, prostate cancer, breast cancer, ovarian cancer, hepatocellular carcinoma, colorectal cancer, endometrial cancer, gastric cancer, EBV-related nasopharyngeal carcinoma, anaplastic large-cell lymphoma (ALCL), colitis-associated carcinoma, bladder cancer and cervical cancer (Figure 3) 6, 8, 36, 38-40. Congruently, TPL2 kinase activity has been implicated in all stages of tumorigenesis, including tumor initiation, tumor promotion and tumor progression; where it modulates cell proliferation, stem cell acquisition, angiogenesis, EMT progression, migration, invasion and metastasis 5, 9.

However, suppressed TPL2 expression is also reported in some cancers 9. For example, reduced TPL2 expression was shown to correlate with poor prognosis and tumor aggressiveness in non-small cell lung cancer (NSCLC) patients 41, 42. Accordingly, TPL2^-/-^ mice exposed to the lung carcinogen urethane demonstrated that loss of TPL2 protein promotes increased cell proliferation and apoptosis resistance due to dysregulated p53 signaling 41. In addition, genetic and epigenetic control mechanisms such as frequency of loss of heterozygosity (LOH) at the TPL2 locus and miR370 upregulation are also associated with TPL2 suppression 41. However, it is still unclear whether these mechanisms directly contribute to TPL2 suppression in lung cancer. This study also proposed that the oncogenic Ras signaling might contribute to TPL2 suppression in lung cancer through the reduction of NF-κB1 p105 protein 41. Consistently, urethane-treated NF-κB1 p105 deficient mice, similar to the TPL2^-/-^ mice, exhibited increased susceptibility to lung cancer that was associated with augmented lung damage, inflammation and K-Ras mutation 43. Furthermore, the reconstitution of TPL2 expression in NF-κB1 p105 and TPL2 deficient mice was shown to inhibit tumorigenesis, possibly by some unknown mechanisms that suppress oncogenic Ras signaling. Correspondingly, these studies highlight the importance of the TPL2 protein in tumorigenesis and pro-tumorigenic inflammation. Moreover, due to its interaction with NF-κB1 p105 and ABIN2 protein, it is possible that altered TPL2 protein expression might contribute to the aberrant activation or suppression of other signaling pathways, thereby promoting pro-tumorigenic inflammation and tumorigenesis. Hence, further studies investigating the distinguished function of TPL2 protein and TPL2 kinase activity in lung cancer and tumorigenesis are necessary.

### Tumor initiation

Tumor initiation is induced by the occurrence of multiple oncogenic alterations, which provide tumor-initiating cells with advantageous proliferation and survival properties 20, 44. The excessive production of reactive oxygen species (ROS) and inflammatory factors induced by harmful chemicals, persistent infection or chronic inflammatory diseases potentiates DNA damage, genomic instability and oncogenic mutation 20. Although the direct contribution of TPL2 to oncogenic mutations has not been reported, this kinase is essential for the production of ROS and the expression of inflammatory factors associated with genomic instability, including TNFα, IL-1β, and COX2 8, 45-47. Circumstantially, TPL2-activated pathways such as NF-κB and AP-1 might also facilitate tumor initiation by inducing the expression of the activation-induced cytidine deaminase (AID), an enzyme that induces genomic instability and increases mutation probability 20. TPL2 activity can also drive tumor initiation by increasing mutagenic targets and the accumulation of oncogenic mutation through the induction of stem cell acquisition, proliferation and survival 7, 19, 48-50.

### Tumor promotion

Tumor promotion is characterized by enhanced cell proliferation and survival of tumor progenitors that contribute to the development of primary tumors 7, 20, 44. TPL2-regulated chemokines and cell cycle proteins including IL-8, CXCR4 and cyclin D1, can promote cancer cell proliferation, cell survival and stem cell acquisition, thereby increasing the number of DNA-damaged cells that accumulate oncogenic mutations 39, 51. This kinase enhances the proliferation of tumor progenitor cells by phosphorylating the G2/M transition mitotic regulator Pin1 that contributes to malignant transformation, tumor growth and aggressiveness 14, 51, 52. Moreover, TPL2-ERK signaling might also mediate the phosphorylation, maturation and trafficking of the tumor necrosis factor α converting enzyme (TACE/ADAM17) 53. This enzyme promotes cancer cell proliferation and malignant transformation by facilitating cytokine (TNFα), growth factor (TGFα) and receptors (TNFR2, IL-R2, EGFR, and HER4) secretion and activation 53, 54. However, it is yet to be determined whether TPL2-regulated TACE is involved in tumor promotion. Similarly, TPL2 fuels major tumor-promoting cytokine signaling through the production of pro-inflammatory cytokines and the activation of several oncogenic transcription factors, including NF-κB, STAT3 and AP-1, which promote cell proliferation and inhibit apoptosis 7, 14, 38, 55.

One mechanism by which malignant cells ensure their survival is by inactivating tumor suppressor function. Cell cycle regulators and tumor suppressors such as p53 and p27^Kip1^ play a critical role in cellular growth signal homeostasis, whereby they regulate cell proliferation through the induction of cell cycle arrest and apoptosis 20. The TPL2 activity in tumor cells favors cell proliferation and survival over apoptosis by negatively regulating the cyclin-dependent kinase inhibitor p27^Kip1^ and the tumor suppressor p53 expression and functional activity 50, 56. In acute myelogenous leukemia (AML) cells, inhibition of TPL2 activity was shown to increase p27^Kip1^ expression and cell cycle arrest, while the overexpression of TPL2 exerted the opposite effect 56. Hence, TPL2-associated tumor cell proliferation might be partly due to its negative regulation of p27^Kip1^. TPL2 activity also facilitates protein phosphatase 2 A (PP2A) binding to p53, which results in its dephosphorization at serine 15 50. The phosphorylation at serine 15 promotes p53 transactivity by facilitating its binding to the co-activator p300, while simultaneously inhibiting p53 degradation by the E3 ligase human double minute 2 (HDM2) 50. Consequently, TPL2 activity might promote malignant cell survival by potentiating p53 degradation and suppressed activity. However, TPL2 protein might positively induce cell cycle arrest and apoptosis by promoting the accumulation of cell cycle inhibitor and pro-apoptotic proteins such as BCL2-associated X protein (BAX), p21 and p53 in a context-dependent manner 41, 57, 58. Knockdown of ATF2, a transcription factor that negatively regulates expression of cell cycle inhibitor p21 and p53, was shown to induce the expression of TPL2, p53 and p21, while simultaneous depletion of ATF2 and TPL2 suppressed p53 and p21 expression 58. The study indicates that the TPL2 protein is involved in the positive regulation of p53 and p21 expression, and the subsequent induction of cell cycle arrest. The TPL2 protein/kinase activity is proposed to modulate p53 expression through its regulation of the nucleophosmin (NPM), a nucleolus protein that negatively regulates of HDM2 41, 57. However, the molecular events linking TPL2 signaling to NPM regulation of HDM2 and p53 stabilization remain elusive and warrant further studies.

### Tumor progression

**Figure 4:**
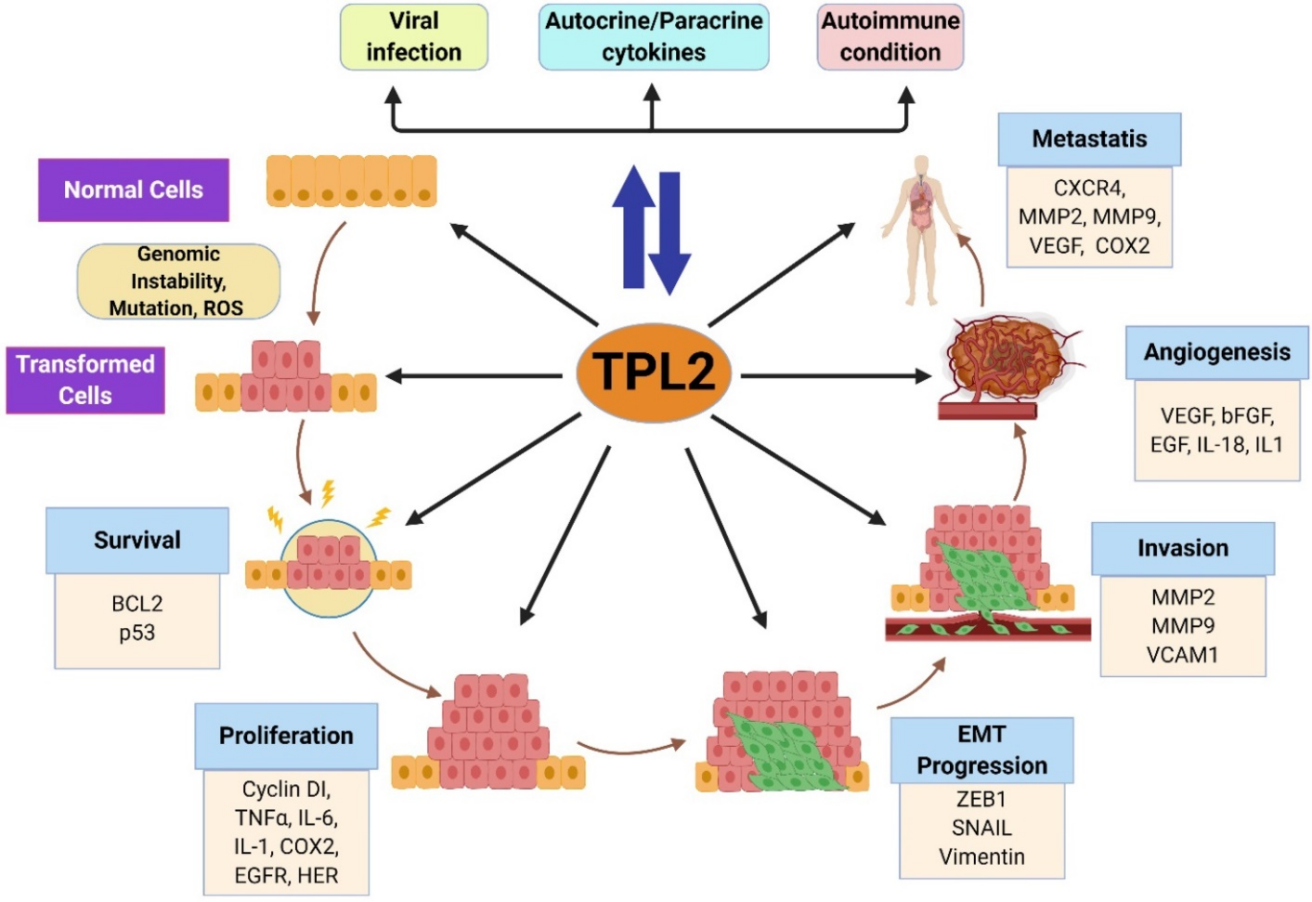
** Roles of TPL2 in tumorigenesis**. Chronic inflammation induced by persistent infection, inflammatory responses and inflammatory diseases activates TPL2 signaling, which subsequently induces the production of pro-inflammatory factors that might contribute to the pro-tumorigenic inflammation microenvironment. Increased TPL2 signaling might promotes genetic instability and mutations through the production of ROS and inflammatory factors. In transformed cells, enhanced TPL2 signaling drives several oncogenic processes: cell survival and proliferation, BCL2, p53, IL-1, IL-6, TNFα, COX2, EGFR and HER; EMT progression, ZEB1, SNAIL and vimentin; invasion, MMP2, MMP9 and VCAM1; angiogenesis, VEGF, bFGF, EGF, IL-8 and IL-1; and metastasis, CXCR4, MMP-9, VEGF and COX2. BCL2: B-cell lymphoma 2; p53: tumor protein p53; IL: interleukin; TNF: tumor necrosis factor; ROS: reactive oxidative stress; COX2: cyclooxygenase; EGFR: epidermal growth factor receptor; HER: human epidermal growth factor receptor; EMT: epithelial-to-mesenchymal transition; ZEB1: zinc finger E-Box binding homeobox 1; MMP: matrix metallopeptidase; VCAM1: vascular cell adhesion molecule 1; VEGF: vascular endothelial growth factor; bFGF: basic fibroblast growth factor; CXCR4: C-X-C motif chemokine receptor 4. (The figure was created with BioRender.com, ©BioRender 2020).

Malignant progression is fueled by enhanced cancer cell proliferation, oncogenic mutation accumulation and suppressed cell apoptosis 20, 44. TPL2 signaling activates transcription factors such as NF-κB, AP-1, STAT3, C/EBPβ, and NFAT that not only contribute to the malignant phenotype, but also trigger angiogenesis, epithelial-to-mesenchymal transition (EMT), and metastasis (Figure 4) 7, 14, 22, 59.

#### Tumor angiogenesis

As the tumor matures, tumor cells secrete various angiogenic factors, such as vascular endothelial growth factor (VEGF), endothelial growth factor (EGF), fibroblast growth factor 2 (FGF2), CXCL1 and IL-8 20. These factors regulate endothelial cell proliferation and tubilization, consequently forming blood vessels that supply nutrients and oxygen to the tumor mass 20. TPL2 also plays a crucial role in growth factor and chemokine-induced tumor angiogenesis. Recently, it was shown that angiogenic factors such as VEGF, EGF and CXCL1 induce TPL2 activation, which in turn modulates endothelial-leukocyte interactions, monolayer permeability and new blood vessel formation through the regulation of Akt, endothelial NOS (eNOS) and ATF-2 60, 61. Moreover, TPL2 serves as a downstream effector of CXCL1, bFGF and EGF, where it activates the transcription factors C/EBPβ, NF-κB and AP-1 that induce VEGF expression, thereby promoting tumor angiogenesis 19, 61.

#### EMT and metastasis

Tumor progression is driven by local invasion and distant metastasis of transformed cells via blood and lymph vessels. Tumor cells acquire their invasive properties through a process known as epithelial-to-mesenchymal transition (EMT), which is characterized by reduced expression of epithelial markers such as E-cadherin. The suppression of E-cadherin results in cell-cell junction detachments, allowing the cells to invade the underlying basement membrane and freely enter the circulation 62. TPL2 is highly expressed in metastatic tumor cells, where it promotes EMT by suppressing E‐cadherin expression while promoting vimentin expression 19, 62. TPL2 also functions downstream TGFβ1 and CXCL12/CXCR4 signaling, leading to the expression of the transcription factors Snail 1 and zinc finger E-Box binding homeobox 1 (ZEB1) that negatively regulate E-cadherin expression 7, 19. The TPL2 regulation of Snail 1, ZEB1, E-cadherin and vimentin underscore its importance in EMT progression and more studies dissecting the mechanisms in which TPL2 regulates these factors are necessary.

TPL2 also mediates the tumorigenic and metastatic potential of cancer cells by inducing the expression of CXCR4 and propagating the FAK/Akt signaling pathways 7, 49, 63. Moreover, the TPL2-ERK1/2 signaling pathway can induce metastasis by activating the transcription factor ETS translocation variant 4 (ETV4) that induces the expression of matrix metalloproteinase-25 (MMP25) 64. MMP25 is a membrane-anchored matrix metalloproteinase that plays a vital role in the onset of tumor dissemination and the induction of the COX2/PGE2 signaling pathway 64, 65. Clinical samples from cancer patients further confirm the connection between TPL2 and metastasis. TPL2 expression and activation correlate positively with distant metastasis and poor prognosis in clear cell renal cell carcinoma (ccRCC) and colorectal cancer, emphasizing the importance of TPL2 in EMT phenotype acquisition and tumor metastasis 40, 49.

## TPL2 and tumor immunity

Since its discovery, TPL2 has emerged as an essential modulator of immune homeostasis and tolerance, immune components generation and function and cytokine-dependent inflammation 1, 9. The balance between the tumorigenic and anti-tumor immunity function of inflammation in the tumor microenvironment is dictated by the abundance and activation state of distinct cell types as well as the expression profile of various immune mediators and modulators 20. The tumor microenvironment consists of a diverse cellular constituent including innate immune cells (macrophage, dendrite cells, neutrophils and natural killer T (NKT) cells), adaptive immune cells (T cells and B cells), mast cells and myeloid-derived suppressor cells (MDSCs) and cancer cells/surrounding stroma (fibroblasts, endothelial cells, pericytes and mesenchymal cells) 20, 66. These heterogeneous cells act in autocrine and paracrine mechanisms to modulate tumor progression and communicate with each other through direct contact or cytokine and chemokine signaling 20, 66. TPL2 is identified as a crucial oncogenic signaling molecule that contributes to immune cell generation, recruitment and function through the modulation of tumorigenic and immune regulating cytokines (Table 2) and chemokines (Table 3) 1, 9.

### Myeloid cells

Tumor-associated macrophages (TAMs) are classified into two reversible phenotypes, M1-type macrophage (also known as classically activated macrophages) and M2-type macrophage (also known as alternatively activated macrophages, AAMs) 20, 44. Functionally, M1-type macrophages have an inflammatory phenotype characterized by the production of a plethora of inflammatory factors, including TNFα, IL‑1β, IL‑6, COX2, IL-12 and iNOS, while M2-type macrophage release immunosuppression factors including IL‑10, TGFβ and arginase 1 (ARG1) 20, 44. Concomitant with their function, the inflammatory M1-type macrophage and anti-inflammatory M2-type macrophage can exert either pro-tumorigenic activity or anti-tumorigenic activity in a context-dependent manner 44, 67.

The activation of the TPL2 kinase activity in TAMs promotes the acquisition of an intermediate M2 macrophage phenotype that promotes inflammation (TNFα, IL‑1β and IL‑6) and immunosuppression (IL-10) while suppressing the production of tumoricidal effectors such as nitric oxide (NO) and IL-12 68, 69. Moreover, blocking of TPL2 activity through gene deletion or pharmacological inhibition was shown to inhibit tumor progression of hematopoietic malignancies and promote tumor shrinkage by suppressing the pro-inflammatory signature of TAMs and increasing the M1 (iNOS^+^)/M2 (Arg^+^) macrophage ratio in a tumor-stage dependent manner 68, 69. Together, these studies identify TPL2 activity as an essential modulator of TAMs polarization and function, and its activity can be targeted to promote macrophage tumoricidal activity. Interestingly, the TPL2^-/-^ macrophage phenotype is similar to that reported in TAMs with conditional deletion of IKKβ and macrophages expressing a dominant-negative IKKβ mutant 70. These studies strongly imply that the previously proposed IKKβ-NF-κB regulation of macrophage polarization in the tumor microenvironment might be partly due to IKKβ-TPL2 signaling 20, 44.

### Lymphocyte

Natural killer (NK) cells are potent anti-tumorigenic cells that kill malignant cells by direct cytolysis or through the production of immunostimulating cytokines, especially in lymphomas and leukemia 44. TPL2 activation might impair NK tumoricidal function in a context-dependent manner by its positive regulation of IFNγ production and its negative regulation of cytokines that modulate NK proliferation and cytolysis function, IFNβ and IL-12 44, 71.

CD8+ cytotoxic T lymphocytes (CTLs) and helper T (Th1) cells are the most prominent and potent anti-tumor immune regulators. TPL2 kinase plays a crucial role in CTLs and Th1 cell development, proliferation, differentiation and effector function downstream of TCR, suggesting that altered TPL2 signaling might compromise T cell-mediated anti-tumor immunity 72-74. However, TPL2 activity is reported to transduce the IL-2 and TNFα proliferative signals in T cells, while constitutively active TPL2 was shown to induce T cell transformation and tumorigenesis, proposing that TPL2 activity might create a feedforward loop that propagates T cell malignancies 48, 75-77. On the contrary, 2C T cell receptor (TCR) transgenic mice crossed with TPL2^-/-^ background exhibited enhanced proliferation and acquisition of an exacerbated effector phenotype leading to the development of CD8+ T cells lymphomas 78. Nevertheless, 2C transgenic mice are reported to be prone to spontaneous T cell lymphoma, while 2C transgene insertion into mutant mice results in accelerated lymphomagenesis 79. Given that TPL2^-/-^ parental mice do not develop lymphomas, increased proliferation induced by loss of TPL2 might synergistically contribute to accelerated lymphomagenesis under pro-tumorigenic conditions 78, 79. Further studies dissecting the exact mechanisms contributing to tumorigenesis in the presence of 2C transgene and TPL2 ablation are required.

At present, the association between TPL2 activity in T cells and tumor immunity remains elusive, and we can only speculate about the mechanisms in which TPL2 activity blocks T cell infiltration and function. For example, TPL2 activity in macrophage and dendrite cells might compromise CTLs and Th1 development and function through its negative regulation of antigen-presenting cells (APCs) phenotype and function 80-82. Furthermore, TPL2-ERK1/2 signaling downstream TCR activation can contribute to T-cell tolerance and dysfunction through its regulation of cytotoxic T lymphocyte antigen-4 (CTLA-4), an immunogenic protein receptor that negatively regulates T cell function 78, 83. Future studies should focus on exploring the role of TPL2 activity in T cell tolerance, immune editing and anti-tumor immunity in the tumor microenvironment.

The inhibitory function of TPL2 activity on regulatory T (Treg) cells development and function can exert either pro-tumorigenic or anti-tumorigenic function in a context-dependent manner 84, 85. In cancers where Treg cells infiltration in the tumor bed is associated with tumor progression and poor prognosis such as breast cancers, cervical cancers and renal cancers, TPL2 activation might promote anti-tumor immunity by inhibiting the generation and suppressor functions of Treg cells 72, 84, 85. However, loss of IL-10 and Treg cells generation was associated with increased intestinal tumorigenesis in Apcmin mutated mice, illustrating the importance of Treg cell generation and function in inflammation-associated tumorigenesis 86.

### Cancer-associated fibroblasts and cancer cells

The tumor microenvironment in mature tumors is characterized by a heterogeneous group of activated fibroblasts known as cancer-associated fibroblasts (CAFs) that interact with cancer cells and other stromal cells to propagate tumor growth and disease progression 44, 87. The TPL2 activity is implicated in CAFs activation, where it sustains the pro-inflammatory transcriptional signature that contributes to tumor progression and metastasis 25, 65. Despite its essential role in the production of inflammatory factors, TPL2 loss in CAFs was reported to promote NF-κB activation, inflammation and tumorigenesis 88. Consistently, cell-specific TPL2 ablation in intestinal subepithelial myofibroblasts (IMFs) and keratinocytes resulted in increased hepatocyte growth factor (HGF, also known as scatter factor) production that was proposed to propagate increased tumor burden in TPL2 deficient mice 89, 90. HGF released by fibroblast exerts its tumorigenic function by activating the tyrosine receptor, c-Met, on tumor cells, which in turn activates various growth factors and signaling pathways involved in cancer cell proliferation, apoptosis resistance, invasion, angiogenesis and drug resistance 88-90.

**Figure 5:**
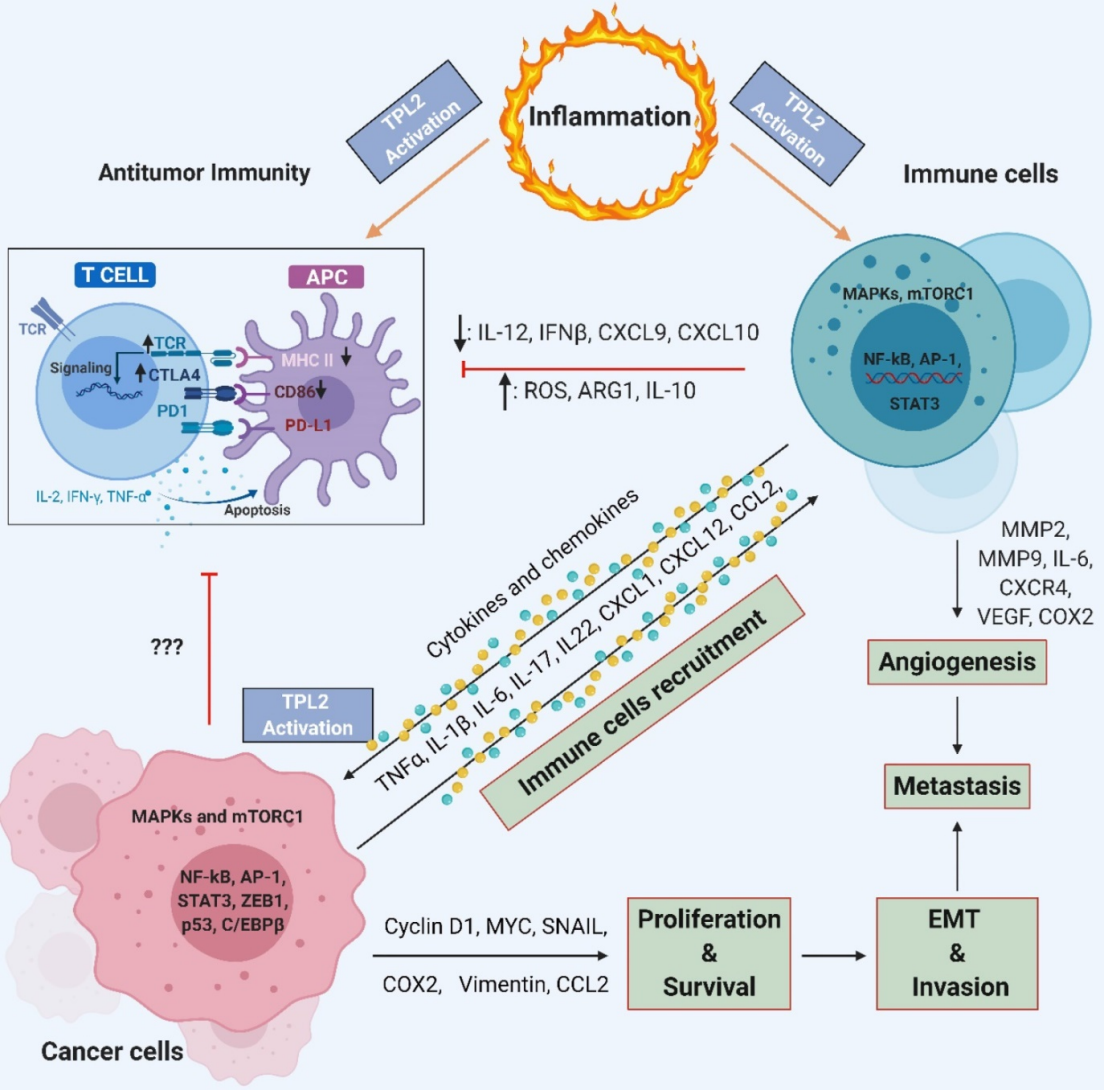
** TPL2 signaling allows crosstalk between immune cells and cancer cells**. TPL2 activation in immune cells induces the production of pro-inflammatory cytokines, chemokines, growth factors and proteinase such as TNF, IL-6, IL-1, CXCR4, VEGF, MMP2 and MMP9. Pro-inflammatory cytokines, chemokines and growth factors activate TPL2 in cancer cells, which then activates signaling pathways such as MAPKs, mTORC1, NF-κB, AP-1, STAT3, ZEB1 and C/EBPβ that promote inflammation, cancer cell proliferation and survival, EMT progression, invasion, angiogenesis and metastasis. Cancer cells can recruit more immune cells to the tumor microenvironment by producing chemokines, thereby creating a chronic feedforward loop that augmenting and maintain the local inflammatory state and promotes tumorigenesis and metastasis. TPL2 activity inhibits the expression of IL-12, IFNβ, CXCL9, CXCL10, MHC-II and CD86 by APCs, thereby preventing their maturation and compromising T-cell antitumor immunity. The production of IL-10, ARG1 and ROS in myeloid cells and cancer cells strengthen the immunosuppressive network, contributing to tumor growth. (IL: interleukin; TNF: tumor necrosis factor; MMP: matrix metalloproteinase; MAPKs: mitogen-activated protein kinases; mTORC1: mammalian target of rapamycin complex 1; NF-κB: nuclear factor kappa-light-chain-enhancer of activated B; AP1: activator protein 1; STAT: signal transducer and activator of transcription; C/EBPβ: CCAAT-enhancer-binding proteins β; VEGF: vascular endothelial growth factor; EMT: epithelial-to-mesenchymal transition; IFN: interferon; CXCL: C-X-C motif chemokine ligand; MHC-II: class II major histocompatibility complex; APCs: cells antigen presenting cells; ARG1: arginase 1; ROS: reactive oxygen species; COX2: cyclooxygenase 2; CD86: cluster of differentiation 86; TCR: T cell receptor; CTLA4: cytotoxic T-lymphocyte-associated protein 4). (The figure was created with BioRender.com, ©BioRender 2020).

The TPL2 kinase activity is proposed to create an inflammatory feedforward loop that mediates the extensive and dynamic crosstalk between cancer and immune cells. This crosstalk contributes to the pro-tumorigenic inflammatory microenvironment that promotes tumor progression and compromised anti-tumor immunity (Figure 5). For example, TPL2-regulated IL-17 and IL-22, which are produced by lymphocytes, were proposed to control cell transformation, cancer cell proliferation and tumorigenesis in breast cancer through the activation of Pin 1, AP-1 and STAT3 in a TPL2-dependent manner 14, 51, 91. Together, these reports identify TPL2 as a crucial oncogenic signaling molecule that contributes to tumorigenesis through the activation of multiple oncogenic pathways, the production of pro-tumorigenic inflammatory factors and the suppression of anti-tumor immunity.

### Immunosuppression and immune evasion

Tumor cells express non-self-antigens that can promote antigen presentation and tumor-killing by NK cells, CTLs, and Th 1 cell, thereby promoting tumor shrinkage 20. Other critical players in the anti-tumor immune machinery include APCs, which present antigens to lymphocytes; Tregs, tasked with modulating immune homeostasis by suppressing immune cell activity; and immunoregulators, such as type I IFN, GM-CSF and IL-12 20. In established tumors, tumor cells manipulate the host anti-tumor immune responses by inhibiting antigen-presentation and establishing an immunosuppressive tumor microenvironment that compromises the anti-tumor activity of lymphocytes 20. Correspondingly, TPL2 kinase activity can facilitate immune evasion mechanisms by impairing APCs' function and promoting an immunosuppressive microenvironment, thereby tilting the balance in favor of pro-tumorigenic immunity over anti-tumor immunity 82, 92, 93.

Macrophages and dendrite cells are professional APCs that play an essential role in modulating anti-tumor immunity through the regulation of Th1 and Treg-mediated immune responses. The APCs function in inducing Th1-mediated tumor immunity is facilitated by their high levels of class II major histocompatibility complex (MHC II), costimulatory molecules, such as CD80 and CD86 on their cell surface and the expression of IL-12 87. Although studies exploring TPL2 kinase function in APCs in the tumor environment are lacking, TPL2 activity in macrophage and dendrite cells is associated with increased inflammation, increased IL-10/IL-12 ratio and suppressed CXCL10, MHC II and IFNβ production 80, 82, 92-94. Consequently, TPL2 activity might lead to the dysregulation of the APCs phenotype and inhibition of the antigen-processing machinery, thereby promoting CD4+ T cells' unresponsiveness. Consistently, the incubation of antigen-specific CD4 (+) T cell with macrophages derived from TPL2^-/-^ mice resulted in enhanced Th1 polarization and IFN-γ production, suggesting that TPL2 activity interferes with the APCs-mediated induction of T cell immune responses 80, 82. TPL2 signaling can further interfere with lymphocyte-mediated anti-tumor immunity by enhancing the infiltration of immunosuppressive cells such as Treg cells and MDSCs through the induction of CCL22, CXCL1 and CXCL2 94-96. Regrettably, there are currently no studies exploring the role of TPL2 activity in APCs, Treg cells and MDSCs cells in the context of tumor immunity and cancer.

In cancer, the immunomodulatory cytokine IL-10 is proposed to induce anti-tumor immune stimulation and tumor-promoting immunosuppression in a context-dependent manner, making it an attractive target for cancer therapy 97. The function of TPL2 activity in immunosuppression is depicted by its role in the regulation of IL-10 signaling and pathogen-induced immunomodulation 92. The thrombocytopenia syndrome phlebovirus non-structural protein (NS) immunosuppression involves its interaction with ABIN2 protein, thereby inducing TPL2 complex formation and signaling activity, which results in enhanced expression of the immunosuppressive cytokine lL-10 98. Moreover, the oncogenic virus human cytomegalovirus (HCMV), associated with increased risk of colorectal cancer, was shown to enhance its immunomodulation potential by encoding the cytomegalovirus-encoded human interleukin-10 (cmvIL-10) 99, 100. The cmvIL-10 is a homolog of human IL-10 (hIL-10) that induces the upregulation of IL-10 in a TPL2-dependent mechanism, thereby implicating TPL2 in pathogen-induced immunomodulation 99, 100.

Together, these studies suggest that TPL2 might contribute to compromised anti-tumor immunity by the subversion of APCs function, and the promotion of immunosuppression mechanisms. Moreover, the function of TPL2 activity in pathogen-mediated immune evasion proposes a possible mechanism in which tumor cells might manipulate the host immune system to promote an immunosuppressive tumor microenvironment.

## TPL2 and its adaptor function

Contrary to the tumorigenic function of the TPL2 kinase illustrated in the above sections, TPL2 ablation is shown to increase tumorigenesis in experimental skin cancer, colitis-associated cancer (CAC) and lung cancer 41, 89, 101. The increased tumorigenesis in TPL2^-/-^ mice is attributed to augmented inflammatory responses due to increased inflammatory cell infiltration and malignant transformation driven by exaggerated NF-κB activation 88, 101. The inhibition of NF-κB with SN50 was shown to suppress inflammatory cell infiltration and NF-κB activity in wild type (WT) mice and keratinocytes 88, 102. Interestingly, the inhibitor was ineffective in blocking the exaggerated NF-κB signaling in TPL2^-/-^ mice and keratinocytes 88, 102. Hence, the loss of TPL2 might contribute to the activation of additional NF-κB activating signaling pathways that are not targeted by the respective inhibitor. These studies underscore the importance of the TPL2 protein in modulating NF-κB signaling and tumor-promoting inflammation.

Moreover, altered COX2 signaling is implicated in driving tumorigenesis in TPL2^-/-^ mice, whereas the COX2 inhibitor, celecoxib, was shown to exert anti-tumor activity in these mice 89, 102. Elsewhere, celecoxib was reported to inhibit cancer stem cell properties of colorectal cancer (CRC) cells by targeting c-Met activity, raising the possibility that celecoxib anti-tumor effect in TPL2^-/-^ mice was as a result of suppressed c-Met activity 103. Consistently, squamous cell carcinomas (SCCs) and keratinocytes from TPL2^-/-^ mice showed increased HGF expression and c-Met activation that contributed to increased stem cell acquisition and metastasis 90. However, pharmacological inhibition of c-Met results in a significant reduction in tumor burden in TPL2^-/-^ mice further implicating c-Met activation in tumor progression in TPL2 deficient mice 89, 90. Loss of TPL2 in IMFs also resulted in the increased production of HGF and tumor susceptibility in TPL2^-/-^ mice 89. Intriguingly, CAC model mice with fibroblast-restricted IKKβ ablation express a similar HGF-dependent phenotype, suggesting the involvement of a common pathway 89, 104. Since IKKβ-NF-κB1 p105 signaling is involved in the activation of the TPL2 kinase and NF-κB pathway, NF-κB1 p105 might be involved in the regulation of HGF production.

Together, these studies provide strong evidence of TPL2 signaling function in tumor-elicited inflammation and tumorigenesis. It is worth noting that TPL2 deficiency on its own is not sufficient to induce tumorigenesis, and TPL2^-/-^ mice tumor susceptibility is dependent on tissue damage, inflammation, NF-κB activation and HGF/c-Met signaling. Thus, it is possible that TPL2 is not a tumor suppressor and its ablation contributes to tumorigenesis under conditions that enhance inflammation and tissue injury. Moreover, given that TPL2 associating proteins, NF-κB1 p105 and ABIN2, are independently implicated in the regulation of NF-κB signaling, inflammation and tumorigenesis, studies exploring their role in TPL2-related inflammation and tumorigenesis are necessary.

### A20-binding inhibitor of NF-κB 2 (ABIN2)

ABIN2 was initially identified as a downstream effector of A20, which exerts an NF-κB inhibitory function by interacting with IKK complex adaptor protein NEMO (IKKγ) and phosphorylated tyrosine kinase Tie2 through its ubiquitin-binding in ABIN and NEMO (UBAN) domain 105, 106. Consequently, ABIN2 dysregulation results in increased inflammation and tumorigenesis in an NF-κB-dependent manner 107. Given that NF-κB1 p105 and TPL2 protein stabilize ABIN2, and the TPL2 kinase activity regulates ABIN2 gene expression, depletion of either TPL2 or NF-κB1 p105 can result in the suppressed expression of ABIN2 43, 108.

TPL2 kinase activity is implicated in many autoimmune disorders including the inflammatory bowel disease (IBD), a chronic inflammatory disorder linked to colorectal cancer (CRC) 34, 109, 110. In IBD, TPL2 gene polymorphism results in increased TPL2 expression and signaling, which then amplifies pattern recognition receptors (PRRs)-mediated activation of ERK, JNK and NF-κB signaling pathways and cytokine production 34, 110. However, mice lacking TPL2 protein in IMFs showed greater susceptibility to chemical-induced colitis due to impaired compensatory proliferation and COX2 signaling 111. The study proposed that the TPL2 kinase activity was responsible for coordinating fibroblast response to injury and governing epithelial tissue homeostasis through the regulation of basement membrane integrity and wound healing 111. However, the real culprit was provided by a study using mice expressing ABIN2 [D310N], an ABIN2 containing a mutation on the UBAN domain that binds to linear and K63-linked polyubiquitin chains 107, 112. ABIN2 [D310N] mice displayed intestinal inflammation and hypersensitivity to chemical-induced colitis, similar to the TPL2 deficient mice phenotype 111, 112. These studies suggest that the ABIN2 signaling might be responsible for maintaining the epithelial layer integrity, as well as regulating cell proliferation and apoptosis.

Although the mechanism in which ABIN2 modulates intestinal inflammation is not yet understood, it is plausible that its anti-inflammatory role involves its interaction with A20 and its regulation of NF-κB activation. Consistently, ABIN2 knock-in mutation that impairs ABIN2 interaction with A20 was shown to augment house dust mite (HDM)-mediated allergic airway inflammation, a phenotype similar to that reported in TPL-2 deficient mice 113, 114. It is therefore proposed that increased inflammation in TPL2 deficient mice is a result of impaired ABIN2-A20 interaction and signaling. The A20-mediated anti-inflammatory activity partially involves the induction of IL-10 expression and Treg cell generation 115, 116. Interestingly, TPL2^-/-^ mice in the Apcmin/+ genetic background demonstrated enhanced intestinal tumorigenesis due to increased inflammation attributed to suppressed IL-10 secretion and Treg cell generation 86. Here, we propose that impaired A20 signaling due to loss of ABIN2 might partially contribute to the suppressed IL-10 expression and Treg cell generation in the TPL2 deficient mice. However, given that TPL2 kinase is a positive regulator of IL-10 expression, it is also possible that loss of TPL2 activity impairs IL-10-mediated anti-inflammatory signaling, resulting in the subsequent increase of intestinal inflammation that drives tumorigenesis 92. The ABIN2 function in tissue homeostasis and anti-inflammatory responses underscore its importance in inflammation, and these functions should be considered when making phenotypic assumptions in TPL2 ablation studies 107, 108. Unfortunately, it is yet to be determined whether the identified function of ABIN2 in inflammation translates to tumorigenesis, especially in tumors expressing suppressed levels of TPL2.

Similar to the TPL2 deficient mice phenotype, NF-κB1 p105 deficient mice show vulnerability to carcinogen-induced lung tumorigenesis and inflammation 43. Lung tumorigenesis in these mice can be rescued through the reconstitution of either the NF-κB1 p105 or TPL2 protein, suggesting that these proteins are required for the suppression of inflammation and tumorigenesis 43. Since altered NF-κB1 p105 and TPL2 protein expression can lead to the suppression or expression of ABIN2, the increased tumorigenesis in NF-κB1 p105 or TPL2 deficient mice might be due to loss of ABIN2 protein 108, 113, 117. Moreover, mutations and epigenetic mechanisms that target NF-κB1 p105 and TPL2 expressions such as K-Ras mutation, frequency of LOH on the TPL2 locus and miR-370, can potentially regulate ABIN2 and NF-κB signaling, subsequently promoting inflammation and tumorigenesis 41, 113. In addition, impaired ABIN2 signaling might also contribute to c-Met signaling in TPL2 deficient mice through the activation of NF-κB, thereby creating a feedforward loop that promotes tumor progression and metastasis 113, 118, 119. Future studies should focus on elucidating the role of ABIN2 signaling in TPL2-mediated tumorigenesis. Studies investigating whether ABIN2 loss contributes to tumorigenesis in the absence of its stabilizing proteins, TPL2 and NF-κB1 p105, are also necessary.

### NF-κB1 p105

**Figure 6:**
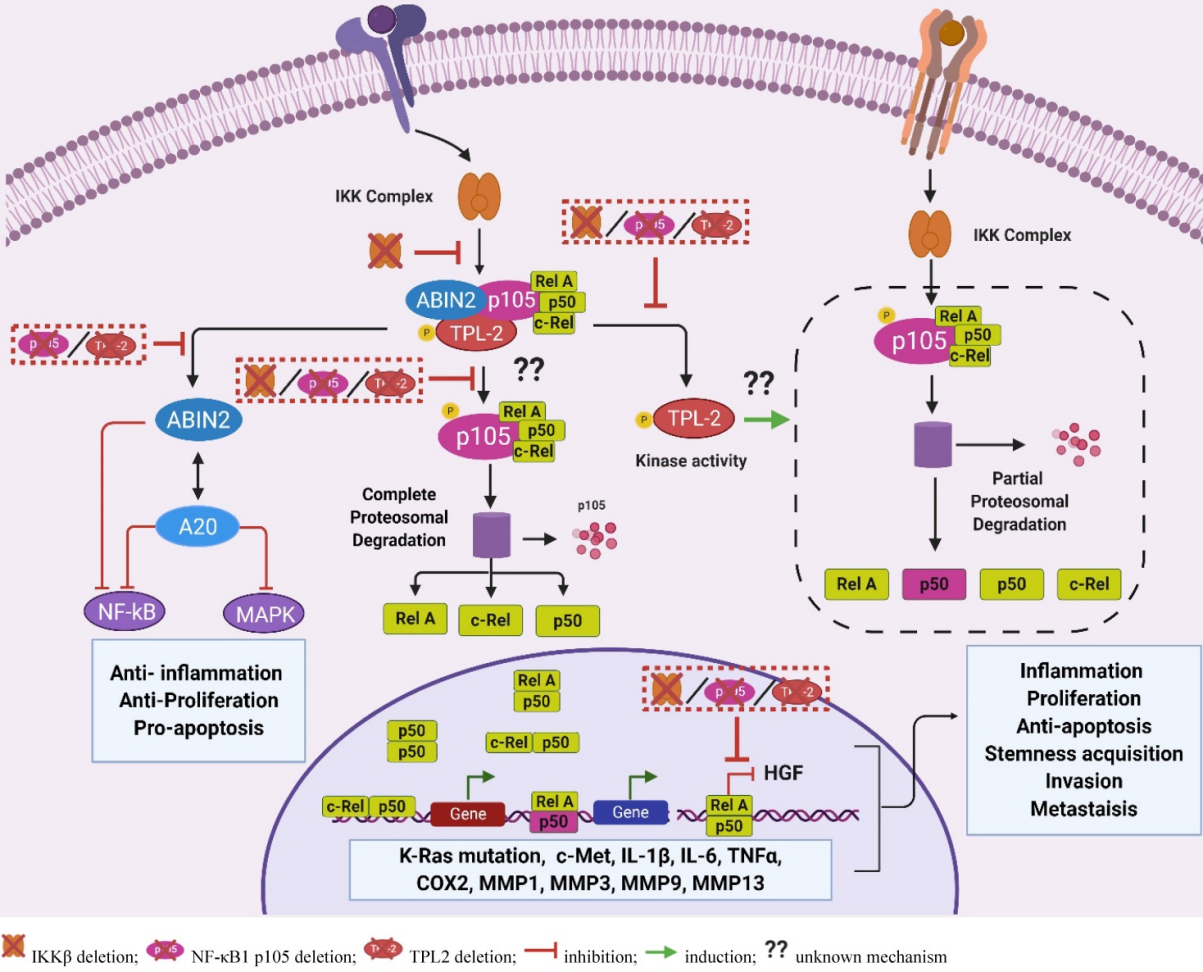
** TPL2 adaptor function.** The TPL2 forms a stoichiometric complex with NF-κB1 p105 and ABIN2. The bound TPL2 phosphorylates the NF-κB1 p105 protein, thereby mediating the complete degradation of NF-κB 1 p105 following TPL2 kinase activation. The complete degradation of NF-κB1 p105 releases the NF-κB1 p105 bound NF-κB subunits including p50, RelA and c-Rel that translocate to the nucleus and regulate gene transcription. Interestingly, activated TPL2 kinase also contributes to the steady state production of p50, and deletion of the TPL2 protein can result in altered p50-dependent signaling. The TPL2 protein also function as an ABIN2 stabilizer. The ABIN2 protein is involved in the negative regulation of NF-κB and positive regulation of the A20 signaling that exerts anti-inflammatory signaling through the inhibition of NF-κB and MAPKs. Protein deletion of either IKKβ, TPL2 or NF-κB1 p105 can result in suppressed RelA signaling leading to increased expression of HGF that propagates tumorigenesis by increasing proliferation, apoptosis resistance, stemness acquisition, angiogenesis and metastasis. Moreover, deletion of TPL2 or NF-κB1 p105 can result in the suppression of ABIN2, leading to increased inflammation resulting from dysregulated NF-κB and MAPKs signaling. Moreover, ABIN2 suppression in the absence of TPL2 or NF-κB1 p105 can result in increased proliferation and apoptosis. NF-κB activity contributes to tumorigenesis by inducing the expression of c-Met, a receptor of HGF; pro-tumorigenic factors including IL1β, IL-6, TNFα and COX2; matrix remodeling enzymes including MMP1, MMP3, MMP9, MMP13. ABIN2: A20-binding inhibitor of NF-κB 2; NF-κB1 p105: nuclear factor kappa-light-chain-enhancer of activated B1 p105; A20: tumor necrosis factor, alpha-induced protein 3; IKKβ: inhibitor of nuclear factor kappa-B kinase subunit beta; HGF: hepatocyte growth factor; MAPKs: mitogen-activated protein kinases; NF-κB: nuclear factor kappa-light-chain-enhancer of activated B; IL: interleukin; TNF: tumor necrosis factor; MMP: matrix metalloproteinase; COX2: cyclooxygenase 2); c-Met: tyrosine-protein kinase Met; K-Ras: Kirsten rat sarcoma). (The figure was created with BioRender.com, ©BioRender 2020).

Generally, the NF-κB1 p105 protein functions as a precursor for the p50 NF-κB subunit. The processing of the NF-κB1 p105 to p50 protein depends on its IKKβ-mediated phosphorylation, which leads to NF-κB1 p105 protein ubiquitination and the proteasomal degradation of its carboxy terminus 120. The NF-κB1 p105 protein also functions as a TPL2 stabilizer and an NF-κB inhibitory protein that retains the p50, RelA and c-Rel NF-κB subunits in the cytoplasm 121. Almost a third of cellular NF-κB1 p105 interacts with the TPL2 protein in cells 121. The interaction between these two proteins promotes the phosphorylation of NF-κB1 p105, thus facilitating its complete degradation in an IKKβ-dependent manner 120, 121. By promoting the degradation of NF-κB1 p105, the TPL2 protein aids in the release and nuclear translocation of NF-κB1 p105-bound p50, c-Rel and RelA, subsequently contributing to the NF-κB signaling (Figure 6) 120, 121.

Interestingly, IKKβ signaling and nucleus RelA signaling are implicated in the suppression of HGF expression through the inhibition of peroxisome proliferator-activated γ (PPAR γ) activity in the HGF gene promoter 122, 123. Transgenic mice and fibroblasts overexpressing the Rel A protein demonstrated suppressed expression of HGF, while RelA inhibition and haploinsufficiency (p65^+/-^) resulted in enhanced HGF expression and c-Met activation in stem cells and macrophages 122, 123. Moreover, genetic deletion or pharmacological inhibition of IKKβ, an upstream activator of RelA and inducer of NF-κB1 p105 degradation, also resulted in the increased HGF expression, which was associated with increased proliferation and tumorigenesis 104, 122. Together, these studies implicate the IKKβ/RelA signaling in the regulation of HGF production. Assuming that suppressed RelA signaling contributes to HGF production in the absence of TPL2, we propose that altered RelA signaling is the common denominator responsible for the phenotypic similarities in both IKKβ-deficient and TPL2-deficient CAFs 89, 90, 104.

The TPL2 protein was also shown to facilitate the p50 nuclear translocation, while the TPL2 kinase activity was proposed to promote the overall rate of p50 production, albeit by an unknown mechanism 121. Moreover, the TPL2 and p50 signaling pathways were independently implicated in the modulation of NF-κB negative feedback mechanisms through the regulation of IkBα and A20 expression 120, 124. Together, these studies suggest that the loss of TPL2 protein might contribute to suppressed p50 signaling, thereby resulting in the reduction of NF-κB antagonists and the subsequent increase in NF-κB-induced pro-tumorigenic inflammation 120-122. Further studies should investigate whether reduced p50 production and the subsequent suppression of NF-κB negative regulators, together with suppressed RelA nuclear signaling contribute to the enhanced NF-κB signaling and HGF production in the absence of TPL2.

## TLP2 and its therapeutic implication

Conventional cancer treatments such as surgery, chemotherapy and radiation exert their anti-tumor effects through the induction of inflammatory responses that promote antigen presentation and adaptive anti-tumor immunity 20, 44. However, therapy-induced inflammation can also have detrimental effects through the stimulation of cancer stem cell acquisition, therapy resistance and tumor recurrence 44. Recently, acquired chemotherapy resistance in response to imatinib in chronic myeloid leukemia model as well as RAF inhibitors chemoresistance in B-RAFV600E melanoma cells was associated with increased TPL2-induced inflammation, highlighting the importance of TPL2 in therapy-induced inflammation and chemoresistance 125, 126. The immunotherapy approach that manipulates the tumor microenvironment to exert anti-tumor immunity has recently attracted much attention in the cancer research community 68, 69. The TPL2 kinase was identified as a promoter of myeloma progression by controlling the inflammatory switch of monocytes/macrophage and M2 polarization 68, 69. In contrast, inhibition of TPL2 through gene ablation or pharmacological inhibitors promoted tumoricidal M1 macrophage licensing and anti-tumor immunity of macrophage-activating immunotherapy in a model of drug-resistant relapsed/refractory myeloma 68, 69. Thus, the inhibition of TPL2 activity can be coupled with chemotherapy and immunotherapy to enhance therapy effectiveness, and to control tumor relapse 56, 69.

Protein kinases contain catalytic domains that share a typical structure and similar mechanism for the transfer of a phosphoryl group from adenosine triphosphate (ATP) onto a bound substrate 127. The binding of ATP with a divalent metal ion such as Mn^2+^, Mg^2+^, Ca^2+^, Fe^2+^, Co^2+^ and Ba^2+^, is an essential step that catalyzes the phosphotransfer process 5, 127. Unlike most kinases that prefer Mg^2+^, the TPL2 kinase prefers Mn^2+^ as the ATP Metal cofactor and has an ATP Michaelis-Menten constant (K_m_) ranging between 20 to 30 μM in the presence of Mn^2+^, suggesting that Mn^2+^ ATP has a high affinity for TPL2 ATP binding site 127. The TPL2 kinase is reported to have distinct structural features that are suggested to favor the development of highly specific, potent and selective TPL2 kinase inhibitors 4, 5. For example, the TPL2 kinase exhibits a relatively low homology to other kinases and contains a proline instead of a conserved glycine on the kinase domain 128. This kinase was also shown to have a more structurally flexible active site with a unique kinase domain fold when complexed with various kinase inhibitors 129. These TPL2 kinase domain properties might contribute to the steep structure-activity relationship demonstrated by various TPL2 kinase inhibitors 129. Moreover, the TPL2 kinase showed an unusually high K_m_ for ATP (ranging between 300-400 μM) in the presence of Mg^2+^ 127. Given that the in vivo potency of kinase inhibitors is dependent on its competitiveness with intracellular ATP, the high ATP K_m_ value in the presence of Mg^2+^ might provide an opportunity for the production of more potent TPL2 kinase inhibitors 5. Multiple chemotypes of ATP-competitive TPL‐2 small molecule inhibitors including 1, 7‐naphthyridine‐3‐carbonitrile, quinoline-3-carbonitriles, indazoles, thienopyridines, and 8‐substituted‐4‐anilino‐6‐amiquionline‐3‐carbonnitrile have been identified by high-throughput screening 130. Testing of these compounds with the structure-activity relationship (RAS) has identified highly specific small molecule inhibitors of TPL2 with low 50% inhibitory concentration (IC_50_) values and high anti-inflammatory properties 5, 130.

TPL2 kinase antagonists such as 4-(3-Chloro-4-fluorophenylamino)-6-(pyridin-3-yl-methylamino)-3-cyano-[1,7]-naphthyridine are proposed to encompass the effects of several anti-inflammatory and anti-tumor agents: anti-cytokine drugs (anti-IL-6, anti-TNFα, anti-COX2, and anti-RANKL); anti-chemokine drugs (anti-CCR2 and anti-CXCR4); and anti-signal inhibitors (anti-NF-κB, anti-STAT3, anti-NFAT and anti-MAPKs) 6, 20, 131. The targeting of individual physiologically functional factors such as cytokine and transcription factor is limited by the requirement of high doses and adverse side effects in patients 20, 44. Hence, the multifaceted effect of TPL2 kinase antagonists provides an attractive approach to combating the dosage-limitation factor and minimizing the diverse effects encountered when targeting specific cytokine and transcription factors. Moreover, the clinically used anti-tumor immunotherapeutic agent IFNα is suggested to exert its anti-NF-κB and anti-COX2 effect by suppressing TPL2 activity, thereby highlighting the importance of TPL2 signaling as an anti-tumor therapeutic target 132. Furthermore, the differential expression of TPL2 in cancer was identified as a prognosis marker in several cancers such as colorectal cancer, non-small cell lung cancer and clear cell renal cell carcinoma (ccRCC) 40, 42, 49. However, there is limited data on the relationship between TPL2 expression/activity and the clinicopathological parameters. Future studies should focus on exploring the mechanisms underlying the differential expression of TPL2 in various human cancers and the correlation between TPL2 expression/signaling and clinicopathological parameters in cancer patients. The TPL2 kinase was also identified as a potential predictive marker of patient responsiveness to MEK antagonist in high-grade serous ovarian carcinoma, underscoring its importance in therapy response 39, 40, 42. Collectively, TPL2 signaling creates a platform to understand disease progression and the mechanism in which other anti-tumor therapies exert their effect; this knowledge can be leveraged to develop more effective and potent drugs.

## Perspective and Conclusion

TPL2 is identified as a molecular linchpin that connects inflammation, tumorigenesis and tumor immunity. The review summarizes the contribution of TPL2 signaling in tumor-promoting inflammation, tumorigenesis, tumor immunity and therapy-induced inflammation. Moreover, the TPL2 signaling is depicted as a multifaceted tool that can be applied as a research target for understanding disease progression, as well as a prognosis and predictive therapy marker, and a therapeutic target in the fight against cancer. In spite of the tremendous progress in the identification of TPL2 activity as a contributor tumor-promoting inflammation and tumorigenesis, there are currently no TPL2 antagonists in the preclinical and clinical trials. Several factors hinder the clinical translation of existing TPL2 kinase inhibitors: 1) lack of TPL2 structural information and fundamental gaps in the molecular mechanism regulating TPL2 complex formation and folding; 2) undefined activity and effectiveness of anti-TPL2 monotherapies and integrative therapies in a multi-metabolic system; 3) lack of molecular biomarkers to predict inhibitor selectivity and monitor patient response to TPL2 kinase inhibitors; 4) limited data on the correlation between TPL2 expression/activity and clinicopathological parameters; 5) cost of current TPL2 inhibitors.

While most evidence supports the oncogenic function of TPL2, several studies have proposed the anti-inflammatory and anti-tumorigenic role of TPL2 signaling. A plausible explanation to this paradox is that in addition to its TPL2 kinase activity, the adaptor function of the TPL2 protein plays an essential role in modulating tissue homeostasis, inflammation and tumorigenesis 107. It would be beneficial to investigate whether ABIN2 and NF-κB1 p105 signaling contribute to the increased NF-κB signaling, COX2 production, and the subsequent activation of the HGF/c-Met signaling in the absence of TPL2. Seeing as some cancers express suppressed TPL2, it will also be crucial to determine whether altered expression of NF-κB1 p105 and ABIN2 are responsible for the decreased TPL2 expression in these tumors. Moreover, micro RNAs such as miRNA-370, miRNA-1180 and miRNA-9, which regulate TPL2, ABIN2 and NF-κB1 p105, respectively, are also implicated in tumor growth and progression 41, 118, 133, 134. We thus propose that identification and analysis of the miRNAs targeting the TPL2 complex subunits might prove essential in deciphering the role of TPL2 signaling in inflammation and tumorigenesis.

Future work should also focus on understanding the complexity and interconnection of the TPL2-NF-κB1 p105-ABIN-2 complex structure, as well as the molecular events that govern TPL2 complex formation and folding 4, 5. The application of biological techniques (such as nuclear magnetic resonance (NMR), X-ray crystallography and cryo-electron microscopy), computational techniques (such as mathematical modeling, homology modeling and threading) and molecular techniques (such as mutagenesis and sequence analysis) would prove essential in exploring the 3D structure and interrelationship of the ternary TPL2 complex. Increased understanding of the ternary TPL2 complex structure can lead to the identification of target sites such as allosteric sites that might lead to the development of novel and more selective non-competitive inhibitors of TPL2 kinase. Moreover, structural-based drug design (SBDD) and ligand-based drug design (LBDD) approaches can be used individually or in combination to identify, optimize and develop more potent TPL2 kinase inhibitors 135, 136. Unlike the SBDD approach that uses established 3D target structures to identify or optimize drug candidates, the LBDD approach involves the identification of structural and physicochemical properties responsible for the drug's biological activity in cases where the target 3D structure is unknown 135, 136. Given the challenges of in vitro production and purification of the TPL2 protein, LBDD approaches might prove beneficial in providing insight on ligand-protein interaction, and the subsequent design and optimization of potent and selective TPL2 kinase inhibitors 127. For example, a three-dimension quantitative structure-activity relationship (3D-QSAR) model generated from a series of quinoline-3-carbonitrile-type TPL2 kinase inhibitors showed that the inclusion of hydrophobic substituents could enhance the TPL2 kinase inhibition activity 137. The use of computation tools might also help cut down the cost of the current inhibitors, subsequently allowing for more* in vivo* experiments. The *in vivo* studies will provide essential pharmacodynamics data and pharmacokinetic properties of the small molecule TPL2 inhibitors in a multi-metabolism system that would contribute to the optimization of TPL2 antagonists for clinical application. The *in vivo* studies will also be crucial in predicting the effectiveness of anti-TPL2 monotherapies and integrative therapies in terms of overall response rate (ORR), progression-free survival (PFS) and overall survival (OS) in various tumors.

In a nutshell, TPL2 activity controls hubs of pro-tumorigenic inflammatory signaling, thereby providing an attractive strategy for targeting inflammatory signaling pathways that curtain both tumor-associated inflammation, tumorigenesis and tumor immunity. However, there are fundamental gaps in our understanding of the TPL2 regulation, signaling and function, which currently makes it difficult to unequivocally determine the overall impact of TPL2 signaling in cancer and tumor inflammatory microenvironment. The complexity and diversity of TPL2 kinase and adaptor function should be considered when selecting the TPL2-blocking therapeutic regimen, and these regimens should be tailored to the patient, cancer phenotype, and influence of the microenvironment. The ideas presented here provide a platform for the development of more integrative and specific anti-inflammatory and anti-cancer therapeutics.

## Figures and Tables

**Figure 1:**
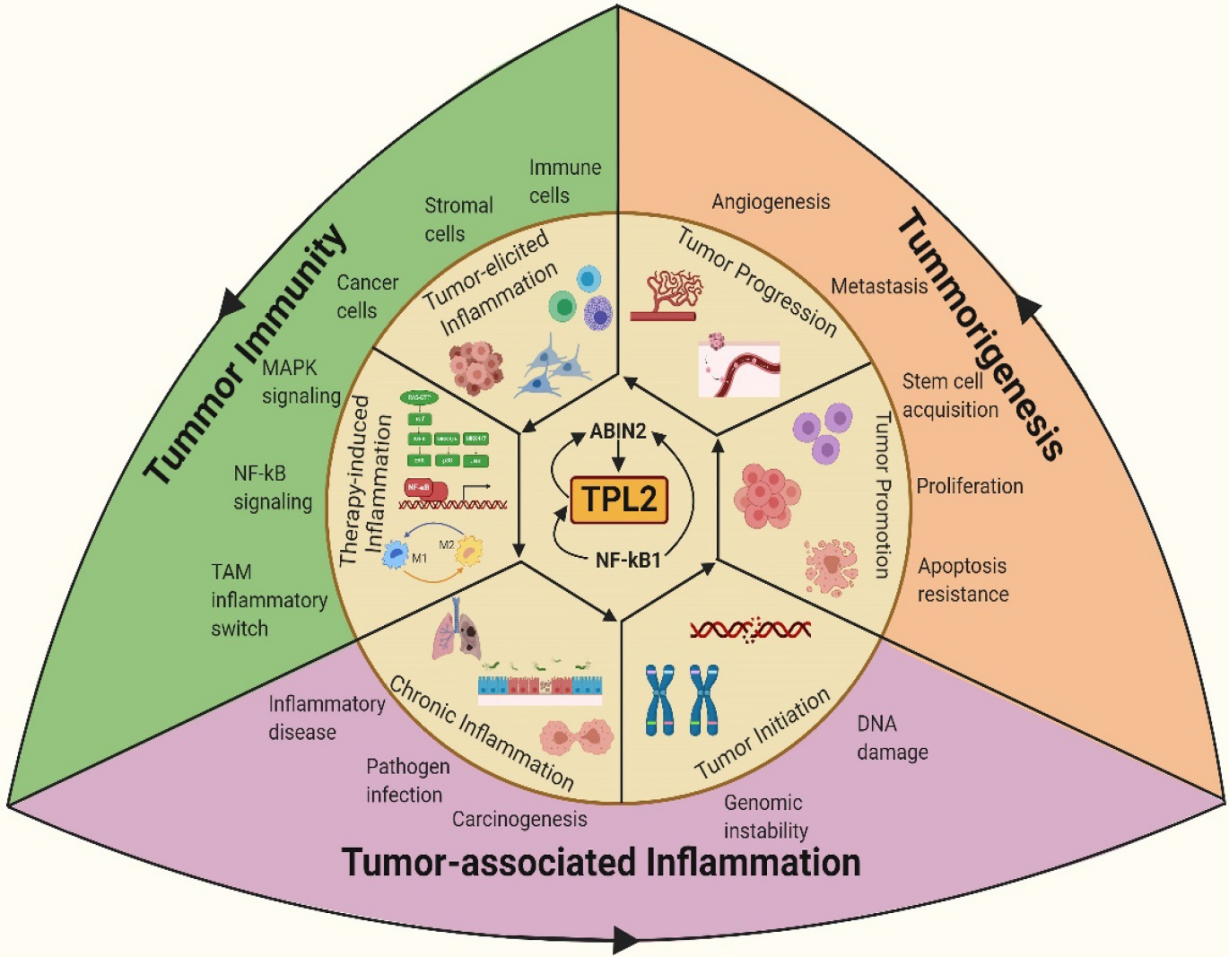
** TPL2 as a molecular linchpin that connects inflammation, tumorigenesis and tumor immunity.** TPL2 activation following underlying inflammatory conditions or as a result of the inflammatory microenvironment during tumor progression promotes tumorigenesis and tumor immunity. (The figure was created with BioRender.com, ©BioRender 2020).

**Table 1:** TPL2 regulation of oncogenic pathways

Signaling Pathway	TPL2 activation mechanism	Function	Reference
MAPKs	MEK1/2 mediated ERK1/2 activation	NF-κB, AP-1, mTORc1, CREB, PDE4D/cAMP signal activation, MSK1 and RSK1 activation, transcriptional and posttranscriptional regulation, immune cell polarization and activation, resistance to RAF inhibition, tumorigenesis	1, 5, 6, 9, 10
	MKK3/6 mediated p38 activation	Inflammatory responses, MSK1 and RSK1 activation, TPL2 stabilization,	1, 6, 9, 10
	MKK4/7 mediated JNK phosphorylation	AP-1 activation, NPM expression and phosphorylation, inflammation, tumorigenesis	6, 9, 10
NF-κB	Direct phosphorylation of NIK TAK1 direct phosphorylation IKKα/β phosphorylation MSK1 phosphorylation of p65 serine 276 IKKβ and RSK1 phosphorylation of p65 serine 536. Rel subunits and p50-Rel heterodimers nuclear translocation following NF-κB1 p105 degradation	Inflammation, cell transformation, proliferation, angiogenesis, apoptosis resistance	6, 9, 44
AP-1	ERK1/2 phosphorylation of c-Fos JNK phosphorylation of c-Jun	Inflammation, cell transformation, proliferation, angiogenesis, apoptosis resistance	6, 9
NFAT	Direct interaction and stabilization of Ca2+/calcinerurin-regulated NFATc protein (NFATc1-NFATc4); NFATc1 nuclear accumulation through activation of Akt/GSK3β signaling	Inflammation, tumor cell proliferation, migration, inhibited apoptosis, osteoclastogenesis	27, 59, 138, 139
STAT3	Induce expression and activation possibly through IL-10, IL-6, IL-17, and IL-33 production	HER2 expression, tumor invasion, angiogenesis, EMT progression and metastasis, IL-10 immunosuppression	14, 98, 99, 140
mTORC1	Akt and ERK1/2 activation	IFNγ production, iNOS suppression and IκBα resynthesize; mRNA stabilization and protein synthesis; inhibit FoxP3 expression	6, 9, 10

**Table 2:** TPL2 regulated cytokines

Cytokine	TPL2 function	Pathways	Tumor-promoting function	Reference
TNFα	Positively regulate transcription; Post-transcriptional regulation. Transducer	NF-κB; AP-1; MAPKs; β-catenin	Inflammation, tumor growth, angiogenesis, EMT progression, immune evasion, chemoresistance	1, 9, 10, 20
IL-6	mRNA stabilization and translation	NF-κB; STAT3; MAPKs; PI3K/Akt; YAP; NOTCH	Cancer-associated inflammation; cancer cell proliferation and survival; stem cell acquisition, angiogenesis, invasiveness; EMT progression and metastasis	18, 20, 143
IFNγ	Optimal induction; Translation; Transducer	JAK-STAT; MAPK; NF-κB	Inflammation, EMT progression, immunosuppression	10, 20, 144, 145
IL-17	Positively regulates transcription; Transducer	NF-κB; MAPKs; STAT3; PI3K/Akt	Inflammation, angiogenesis, metastasis, immunosuppression	6, 10
IL-33	Transducer	NFκB; MAPKs; AP-1; STAT3	Cell transformation, EMT progression, tumor growth	44, 140
IL-10	Positively regulate transcription	JAK-STAT3; NF-κB	Immunosuppression	20, 92, 99
IL-12	Negative regulator		Anti-tumor immunity, inhibit angiogenesis	82, 146, 147
IL-23	Positively regulate transcription	NF-κB; MAPKs; STAT3; PI3K/Akt	Pro-tumorigenic inflammation, tumor growth	148, 149
IFNβ	Suppresses expression; Transducer.	STAT1/2; MAPKs; PI3K/Akt	Anti-tumor immunity, anti-angiogenesis, suppress EMT progression, suppress cancer stem cell properties	93, 150-152

**Table 3:** TPL2-regulated chemokines

Chemokine/Chemokine receptors	TPL2 regulation	Pathways	Tumor-promoting	Reference
CXCL1	Transducer	MAPKs; C/EBPβ; NF-κB; AP-1	Cancer cell proliferation, chemotactic motility and migration, angiogenesis, metastasis; Immunosuppression	61, 153
CXCL2 (MIP-2)	Positively regulate transcription	MAPKs; NF-κB	cell proliferation, cancer cell stemness, immunosuppression, metastasis	12, 154, 155
CXCL3 (MIP-2 β)	Positively regulate transcription	MAPKs; PI3K/Akt	Cancer stem cell maintenance, cancer cell proliferation, migration	12, 154, 156
CXCL8 (IL-8)	Positively regulate transcription; Posttranscriptional modifications	NF-κB; MAPKs; AP-1; PI3K/Akt	Cancer cell proliferation, survival and stemness, angiogenesis, migration, EMT progression and metastasis	23, 157
CXCL12 (SDF-1)	Transducer	MAPKs; STAT3; NF-κB; PI3K/Akt	Tumor growth, angiogenesis, migration, metastasis, chemoresistance	158 7
CCL2	Positively regulate transcription	PI3K/Akt; STAT3; MAPKs; SMAD3; NOTCH1	Tumor growth and migration, angiogenesis, metastasis and invasion, stem cell acquisition, immunosuppression	159, 160
CCL5	Negatively regulate transcription	PI3K/Akt; NF-κB STAT3; MAPKs; mTOR	Cancer cell proliferation, malignant transformation; T cell-mediated antitumor immunity	12, 154, 161
CCR5	Maintain expression; Posttranscriptional modification	MAPKs; NF-κB	Inhibit tumor growth, optimize anti-tumor response, immunosuppression	162, 163
CCL7	Positively regulate transcription	MAPKs	EMT progression, metastasis, tumor growth, promote antitumor immunity	12, 154, 164
CXCL10	Negatively regulate transcription		Patient survival, anti-proliferation, anti-angiogenesis, anti-tumor activity	12, 154, 165
CXCR4	Positively regulate transcription	MAPKs; FAK; PI3K/Akt; NF-κB; NOTCH	Tumor growth, angiogenesis, EMT and metastasis, inflammation, stem cell acquisition	7, 166

## Abbreviations

TPL2: tumor progression locus 2
Cot: cancer osaka thyroid
CXCR: chemokine receptor type
CXCL: C-X-C motif chemokine ligand
HCC: hepatocellular carcinoma
IL-1: interleukin
MCP-1: monocyte chemoattractant protein-1
IFN: interferon
COX-2: cyclooxygenase-2
NF-κB: nuclear factor kappa-light-chain-enhancer of activated B cells
cAMP: cyclic adenosine 3,5-monophosphate
EMT: epithelial-mesenchymal transition
PAMPs: pathogen associated molecular patterns
DAMPs: damage associated molecular patterns
TNFR: tumor necrosis factor receptor
IL1R: interleukin 1 receptor
TLRs: toll-like receptors
ABIN-2: A20 binding inhibitor of NF-κB 2
A20: tumor necrosis factor, alpha-induced protein 3**
IKK: inhibitor of nuclear factor kappa-B kinase
MAPKs: mitogen-activated protein kinases
ERK1/2: extracellular signal-regulated kinases 1 and 2
p38α: MAPKs p38-alpha
JNK: c-Jun N-terminal kinase
TNF: tumor necrosis factor
IL: interleukin
CCL: C-C motif chemokine ligand
RANTES: regulated upon activation, normal T cell expressed and presumably secreted
VEGF: vascular endothelial growth factor
MMP: matrix metalloproteinase
AP1: activator protein 1
STAT: signal transducer and activator of transcription
C/EBPβ: CCAAT-enhancer-binding proteins-β
iNOS: inducible nitric oxide synthase
MHC-II: class II major histocompatibility complex
CRC: colorectal cancer
PRRs: pattern recognition receptors
IMFs: intestinal subepithelial myofibroblasts
CAC: colitis-associated colon cancer
HGF: hepatocyte growth factor
CAFs: cancer-associated fibroblasts
Th: T helper
CTL: cytotoxic T lymphocytes
CCR: C-C chemokine receptor
Th1: T helper 1
ROS: reactive oxygen species
EGF: epidermal growth factor receptor
TGF: transforming growth factor
